# High prevalence of hepatitis B virus susceptibility among persons undergoing community-based hepatitis C virus treatment

**DOI:** 10.1186/s12954-024-00942-x

**Published:** 2024-01-29

**Authors:** Catherine Campusano, Rachel Kanner, Claire McDonell, Meghan Morris, Maria Duarte, Jennifer C. Price

**Affiliations:** 1 Department of Medicine, School of Medicine, University of California, San Francisco, CA USA; 2 Department of Epidemiology and Biostatistics, University of California San Francisco, San Francisco, CA USA; 3 Liver Center, University of California San Francisco, San Francisco, CA USA; 4 Northwestern University Feinberg School of Medicine, Chicago, IL USA

## Abstract

**Background:**

Due to shared modes of transmission, coinfection with hepatitis B virus (HBV) and hepatitis C virus (HCV) is common, and HBV vaccination is recommended for all persons with HCV who remain susceptible to HBV. To identify potential gaps in HBV vaccination among this high-risk population, we aimed to determine the patterns of HBV susceptibility in persons undergoing community-based HCV treatment.

**Methods:**

We performed a cross-sectional study within two community-based HCV treatment programs in an urban US setting. Participants were identified for HCV screening and confirmatory testing via street-outreach recruitment directed at persons experiencing homelessness and currently using drugs. Participants were excluded if HBsAg was reactive. Cohort characteristics were obtained via intake surveys and descriptive analysis was performed by exposure status.

**Results:**

Among 150 participants without chronic HBV receiving community-based HCV treatment, 43% had evidence of prior HBV infection, 26% were immune from vaccination, and 31% were non-immune. Among the subset of the cohort reporting current injection drug use (IDU) (*N* = 100), 31% (*n* = 10) of those aged 24–40 and 47% (*n* = 23) of those aged 41–57 remained susceptible to HBV infection. By contrast only two participants aged 58–74 were HBV non-immune (11%), with 84% immune due to prior exposure.

**Conclusions:**

Our data reflect a high prevalence of HBV susceptibility among persons undergoing community-based HCV treatment. Although younger patients were more likely to be immune due to vaccination, a high proportion remained non-immune to HBV, particularly among those reporting current IDU. Our data reflect a gap in HBV vaccination among younger persons with HCV and suggest a potential role for co-localizing HBV vaccination with community-based HCV screening and treatment.

## Background

Worldwide, over 250 million people are living with chronic hepatitis B virus (HBV) and 70 million with chronic hepatitis C virus (HCV) [1]. In the USA, an estimated 862,000 people are living with HBV and 2.4 million are living with HCV [2, 3]. HBV and HCV coinfection is associated with higher risk of cirrhosis and decompensated liver disease [4] and is more prevalent in populations with risk factors for transmission of both viruses, such as people who inject drugs. Despite a safe and effective vaccine to prevent HBV infection, 26% of infants born in the USA in 2017 did not receive the recommended hepatitis B vaccine at birth [5].

Acute HBV infections are increasing in the USA: the Centers for Disease Control and Prevention reported an 11% increase in new HBV cases from 2014 to 2018 [6]. The rise in HBV cases is highest in regions characterized as epicenters of the opioid crisis, with injection drug use (IDU) identified as a high-risk factor for HBV transmission [6]. These regions are also experiencing increases in acute HCV, highlighting the importance of collaboration and coordination across viral hepatitis programs and activities to address both viral hepatitis epidemics [5], as more than 80% of new HCV infections have been associated with IDU [7]. Additionally, although direct-acting antivirals (DAAs) to treat HCV can achieve sustained virological response, or cure, in nearly all patients who complete a treatment course, 29 unique reports of HBV reactivation were reported to the US Food and Drug Administration between 2013 and 2016 [8]. This underscores the importance of understanding and addressing HBV susceptibility in populations receiving DAA HCV treatment. We aimed to characterize HBV serologies among a cohort of patients receiving community-based HCV treatment outside of a traditional brick-and-mortar clinic. Our overall purpose was to identify modifiable gaps in HBV prevention among this high-risk population.

## Methods

We performed a cross-sectional study within two community-based HCV treatment programs: the UCSF DeLIVER Care van and the No One Waits (NOW) study (NCT03987503). The DeLIVER Care van is a mobile van offering HCV screening and low-threshold HCV treatment which parks outside various service organizations (e.g., methadone clinics, supportive housing) in San Francisco. The NOW study is a clinical trial investigating a point-of-diagnosis HCV treatment model located at a non-clinical community site in San Francisco [9]. Both programs (DELIVER Care and NOW) identified participants for HCV screening and confirmatory testing from street-outreach recruitment targeting people experiencing homelessness and people who use drugs in both treatment programs. HCV screening was performed using the OraQuick HCV rapid antibody test (OraSure Technologies, Inc., Bethlehem, PA), followed by venipuncture and confirmatory HCV RNA PCR testing using real-time PCR (Quest Diagnostics, Secaucus, NH) if reactive. Individuals with HCV RNA viremia were offered HCV treatment. NOW participants with self-reported chronic HBV or with positive hepatitis B surface antigen (HBsAg) were not eligible for HCV treatment through the study, which followed a modified AASLD-IDSA HCV simplified HCV treatment algorithm. For the current analysis, DeLIVER Care patients who were HBsAg positive were excluded. If not previously performed, hepatitis B surface antibody (anti-HBs), and hepatitis B core antibody (anti-HBc) were obtained prior to HCV treatment initiation. Hepatitis B serologies were performed with enzyme immunoassays (EIAs) at local CLIA-approved laboratories. The NOW study and retrospective review of patients living with HCV on DELIVER Care were approved by the Institutional Review Board of the University of California San Francisco.

HBV serologies were used to determine HBV exposure: (1) non-immune (non-reactive HBsAg, anti-HBc, and anti-HBs); (2) immune due to prior infection (non-reactive HBsAg, reactive anti-HBc, ± reactive anti-HBs); or (3) immune due to vaccination (non-reactive HBsAg and anti-HBc and reactive anti-HBs). This study characterized isolated anti-HBc as prior exposure. Anti-HBs results that were equivocal, or borderline, were considered negative.

Demographic data were obtained from screening intake surveys. Liver fibrosis stage was estimated using the Fibrosis-4 index (FIB-4 scores) [10]. Fibrosis was categorized as minimal (FIB-4 < 1.25), moderate (FIB-4 1.25–3.25), or advanced/cirrhosis (FIB-4 > 3.25). Recognizing that both HBV exposure and prior vaccination may vary by age, we stratified our cohort into age tertiles: 24–40 years, 41–57 years, and 58–74 years.

We performed descriptive analysis comparing demographic and other characteristics by HBV exposure group using the Chi-square test for categorical variables. Analyses were performed using Stata/BE software, version 17.0 (StataCorp LLC, College Station, TX).

## Results

A total of 150 participants starting HCV treatment were included in our study: 63 from the DeLIVER Care Van and 87 from the NOW Study. Median age of the cohort was 51 years [IQR 61–41], 69% were male, and self-identified race/ethnicity was 51% White, 27% Black, 11% Hispanic or Latino, and 11% Other (Table 1).

**Table 1:** Demographic factors for general cohort and by HBV exposure status groups (*N* = 150)

	Cohort Demographics n (col %)	Demographics by HBV Exposure Status n (row %)			
	Total cohort (*N* = 150)	Not immune (*N* = 46)	Prior exposure (*N* = 65)	Immune due to vaccination (*N* = 39)	*p*-value
Age					
24–40	36 (24%)	12 (33%)	4 (11%)	20 (56%)	< 0.001
41–57	60 (40%)	26 (43%)	24 (40%)	10 (17%)	
58–74	54 (36%)	8 (15%)	37 (69%)	9 (17%)	
Race					
White	77 (51%)	23 (30%)	32 (42%)	22 (29%)	0.55
Black or African American	40 (27%)	9 (23%)	22 (55%)	9 (23%)	
Hispanic or Latino	17 (11%)	7 (41%)	6 (35%)	4 (24%)	
Other	16 (11%)	7 (44%)	5 (31%)	4 (25%)	
Sex at birth					
Male	104 (69%)	34 (33%)	41 (39%)	29 (28%)	0.35
Female	46 (31%)	12 (26%)	24 (52%)	10 (22%)	
Housing Status, current					
Stable	74 (49%)	19 (26%)	39 (53%)	16 (22%)	0.07
Unstable	76 (51%)	27 (36%)	26 (34%)	23 (30%)	
Lifetime injection drug use					
Yes	137 (91%)	43 (31%)	60 (44%)	34 (25%)	0.55
No	13 (9%)	3 (23%)	5 (38%)	5 (38%)	
Current injection drug use					
Yes	100 (67%)	35 (35%)	37 (37%)	28 (28%)	0.08
No	50 (33%)	11 (22%)	28 (56%)	11 (22%)	
MSM					
Yes	16 (11%)	4 (25%)	5 (31%)	7 (44%)	0.23
No	134 (89%)	42 (31%)	60 (45%)	32 (24%)	
HIV-positive					
Yes	6 (4%)	0 (0%)	3 (50%)	3 (50%)	0.19
No	143 (96%)	45 (31%)	62 (43%)	36 (25%)	
On methadone/suboxone					
Yes	53 (35%)	18 (34%)	24 (45%)	11 (21%)	0.54
No	97 (65%)	28 (29%)	41 (42%)	28 (29%)	
FIB-4 Score					
< 1.25	88 (59%)	32 (36%)	28 (32%)	28 (32%)	0.02
1.25–3.25	50 (33%)	12 (24%)	29 (58%)	9 (18%)	
> 3.25	12 (8%)	2 (17%)	8 (67%)	2 (17%)	

Data among adults aged 24–74 years across three exposure status groups and the general cohort (*N* = 150). *Notes*: The “Other” category encompasses, Alaska Native, American Indian, Asian, Pacific Islander, Native Hawaiian, mixed, or declined to answer on treatment intake surveys. Current housing status, stable, referred to rent, own, SRO, or hotel survey responses. Unstable housing self-reporting referred to treatment or transitional housing, staying with a friend, shelter, outdoors, or in a vehicle responses. FIB-4 scores less than 1.25 indicated minimal fibrosis, 1.25 to 3.25 moderate fibrosis, and greater than 3.25 advanced fibrosis/cirrhosis. HIV serological data were only available for 149 of the 150 cohort

Fifty-one percent of the cohort had unstable housing, which was defined as treatment or transitional housing, staying with a friend, shelter, outdoors, or in a vehicle. The vast majority (91%) reported a history of lifetime IDU, and 67% reported current IDU. Eight percent had suspected cirrhosis based on FIB-4 scores.

Overall, 46 participants were HBV non-immune (31%), 65 were immune due to prior infection (43%), and 39 were immune due to vaccination (26%). Of participants with immunity due to prior infection, 23 had isolated anti-HBc positivity (35%). Among those with isolated anti-HBc, 65% (*n* = 15) were over 57 years old and 30% (*n* = 7) were aged 41–57, while one participant aged 24–40 exhibited the serological profile.

**Fig. 1:**
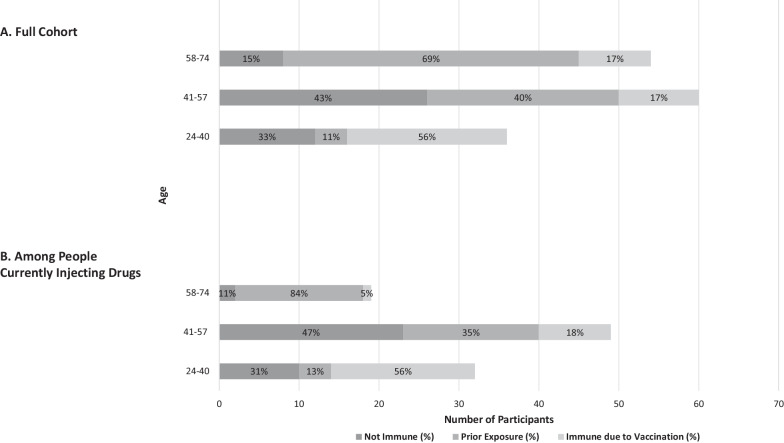
HBV Exposure Status by Age in **A** Full Cohort (*N* = 150), and **B** People Currently Injecting Drugs (*n* = 100). **A** Distribution of HBV exposure status by the three age categories, in the full cohort by number of participants in each category. **B** A subset of the cohort who self-reported current IDU. Distribution signifies HBV exposure status rates in subset for the three age categories. Number of participants in each category is signified by y-axis, this number as a percent of the cohort and subset, age category, is contained in each bar

Immune status varied significantly by age: older participants (i.e., aged 58–74 or 41–57) were more likely to be immune due to prior exposure compared to younger patients (i.e., aged 24–40) (69% and 40%, compared to 11%) and less likely to have immunity due to vaccination (17% and 17%, compared to 56%) (*p* < 0.001) **(**Fig. 1**).**

Because current IDU is a risk factor for HBV transmission, we evaluated HBV exposure status among the subset of the cohort reporting current IDU (*N* = 100). Among these participants, 31% (*n* = 10) of those aged 24–40 and 47% (*n* = 23) of those aged 41–57 remained susceptible to HBV infection (i.e., non-immune) (Fig. 1). By contrast only two participants aged 58–74 were HBV non-immune (11%), with 84% immune due to prior exposure. The younger participants were more likely to have immunity due to vaccination: 56% of those aged 24–40 versus 18% of those aged 41–57 and 5% of those aged 58–74.

## Discussion

We found a high prevalence of prior HBV exposure and a relatively low prevalence of HBV immunity due to vaccination among HBsAg-negative persons undergoing community-based HCV treatment when compared to the general population [11, 12]. Although younger participants were more likely to be immune due to vaccination, a high proportion remained non-immune to HBV, including those at an elevated risk of HBV transmission due to current IDU.

Overall, nearly half of our cohort (43%) had evidence of prior HBV exposure and rose to 69% when those aged 58–74 were isolated. The 2001–2016 National Health and Nutrition Examination Survey (NHANES) data estimated a 20% prevalence of anti-HBc positivity among adults aged 20–59 with any history of IDU [11]. NHANES, however, is limited in its ability to reach those who were not stably housed. Our study population was older, 51% reported unstable housing, and was comprised entirely of people living with HCV. Thus, the high anti-HBc positivity in our cohort is not surprising. As expected, we found that older participants were more likely to have anti-HBc positivity, with a striking 84% of those over 57 who reported current IDU having anti-HBc positivity.

The first plasma-derived hepatitis B vaccine licensed for use in the USA in 1981, and starting in 1991, all infants and young children were recommended to receive it [13]. Therefore, it is unsurprising that our younger participants born after this approval were most likely to have immunity due to prior vaccination, at 56% in the full cohort and among those currently injecting drugs aged 24–40. However, our oldest participants were less likely to have immunity due to vaccination- only 17% in the full cohort and 5% among those currently injecting drugs. By comparison, the 2021 National Health Interview Survey (NHIS) found that 20% of adults for ≥ 60 years reported HBV vaccine receipt [12]. However, we did not collect information on prior vaccination status and therefore could not definitely determine whether susceptibility to HBV was due to non-vaccination versus lack of seroprotective response or waning immunity to prior vaccination.

The most notable finding in our study was the high rate of HBV susceptibility among younger participants in our cohort, despite vaccine availability (33% for those aged 24–40). This is particularly important because most study participants reported current IDU (67%), and all were at elevated risk of adverse HBV-related liver outcomes due to chronic HCV. Our data demonstrate a gap in HBV prevention among high-risk individuals but also identify a potential opportunity to leverage resources combatting HCV to prevent new HBV infections. Our cohort was comprised of participants in the NOW Study and UCSF DeLIVER Care van, two initiatives aimed to eliminate barriers to HCV treatment by pairing community-based HCV testing services with low-threshold HCV treatment outside brick-and-mortar clinics. High HBV susceptibly trends for this population suggest a potential role for co-localizing HBV vaccination within the community-based HCV screening and treatment model. This approach is particularly appealing to reach young people who inject drugs—who are less likely to seek healthcare in traditional settings outside of emergency services [14]–and remain at highest susceptibility to HBV [15].

## Limitations

The findings of this cross-sectional study, particularly the HBV exposure rates of this population, should be weighed in the context of absence of HBsAg-positive participant data. We only excluded one participant with HBsAg positivity, but individuals with known chronic HBV were not eligible for HCV screening in the NOW study. Another important limitation, as noted above, is our lack of prior vaccination history. Finally, we also considered isolated anti-HBc positivity to be evidence of prior exposure even though we could not rule out false positivity, although false-positive anti-HBc is rare in a population with risk factors for HBV exposure [16].

This study was conducted in a city with a strong focus on harm-reduction services, facilitating interaction with a patient population who are less likely to seek care, to ultimately offer low-threshold HCV treatment for our study population. Although feasibility of recruitment of this participant demographic may not be generalizable to other geographic locations, our findings reflect a gap in HBV vaccination among high-risk individuals. Outlined in the US National Hepatitis C Elimination Program [17], increased public health capacity to address HCV may offer the opportunity to implement co-localization of HBV vaccination in community-based treatment settings in other cities.

## Conclusions

In summary, in our cohort of adults with history of IDU who underwent HCV screening and treatment outside of a traditional health care setting, we found a high prevalence of prior HBV exposure among older adults and a high prevalence of HBV susceptibility among younger adults. Co-localizing HBV vaccination should be considered in programs designed to offer low-threshold HCV treatment in community-based settings.

## Data Availability

The dataset used is available from the corresponding author upon reasonable request. CC had full access to all the data in the study and takes responsibility for the integrity of the data and the accuracy of the data analysis.
